# Mental preparation of karateka for sports competition in kata

**DOI:** 10.3389/fspor.2024.1525853

**Published:** 2025-01-09

**Authors:** Paweł Adam Piepiora, Julia Barbara Jurczyk, Jolita Vveinhardt

**Affiliations:** ^1^ Faculty of Physical Education and Sports, Wroclaw University of Health and Sport Sciences, Wrocław, Poland; ^2^ Faculty of Social Sciences and Humanities, Witelon State University of Applied Sciences in Legnica, Legnica, Poland; ^3^ Institute of Sport Science and Innovations, Lithuanian Sports University, Kaunas, Lithuania

**Keywords:** combat sport, Kyokushin, martial art, Olympic karate, Shotokan

## Abstract

Mental preparation for sports competition in karate is significant, as it is deeply embedded in the philosophical and ethical values that underpin this combat method. In practice, the mental preparation of karateka varies depending on the type of competition, for example preparation for kata (forms) and kumite (fights). Thus, this perspective offers a concise account of the authors' viewpoint on the leading mental skills required of kata competitors. It is argued that self-esteem, inner speech, imagination, visualisation, values and personality play a significant role in the development of confidence. In addressing stress, attention was directed towards the role of arousal, coping strategies, Jacobson's progressive relaxation, Schultz's autogenic training, biofeedback, schemas and scripts. Effective management of anxiety relies on the utilisation of mindfulness and desensitisation techniques. In maintaining attention, effective attention management, attention styles, the ability to filter out distractions, mantras and affirmations are significant. Conversely, practicing tasks that induce a Stroop effect can improve executive function skills. The above elements of mental preparation for kata competitors are universally applicable to all competitors in this field, yet they are not the sole elements that may be employed. Given the individual predispositions of kata competitors, other mental training techniques may also be applicable.

## Introduction

The twenty principles of karate, devised by Gichin Funakoshi, suggest that mental training is of greater importance than physical training (1). This implies that karate competitors, prior to engaging in the competitive aspects of kata (forms) or kumite (fights), are already engaged in a process of mental preparation to overcome their own weaknesses and limitations. The fundamental tenet of karate philosophy and ethics is the process of self-improvement. This is evidenced by research findings indicating a correlation between karate training and the development of mental abilities such as self-confidence, self-control (2), executive functions (3), aggression management (4), concentration (5), and resilience (6). A successful karateka is characterised by low levels of aggression, high levels of extraversion, a need for continuous experience and a low sense of anxiety (7). Moreover, the cultivation of selected psychological competencies yields enhanced performance outcomes. Mental resilience and self-efficacy exert a significant influence on the acquisition of specific fighting techniques (8). The emotional aspect is also important in karate (9–11). Additionally, high cognitive functioning − sustained attention and reaction time − distinguishes competitive karateka from those engaged in other combat sports (12). Consequently, karate can be regarded as a method of combat, whereby systematic physical activity is designed to cultivate heightened concentration, stress management abilities, elevated emotional resilience and self-control skills (13).

**Figure 1:**
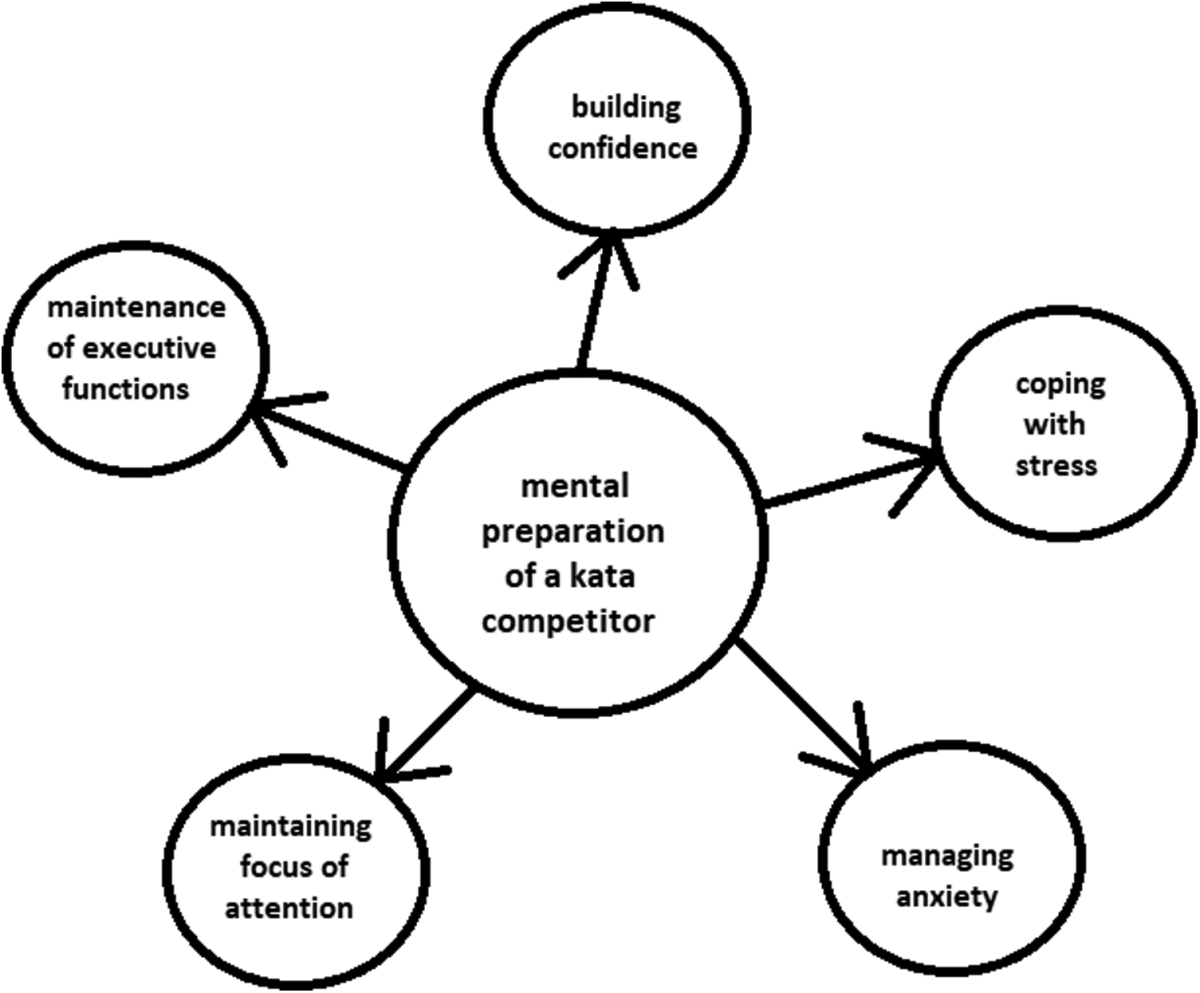
Mental preparation of a kata competitor.

In addition to the research reports described above, the experiences of sport psychologists working with karateka on mental preparation are of significant value. By drawing on these experiences, one may identify the key mental skills that are important for both coaches and karateka (14). Accordingly, this perspective represents a concise presentation of the authors' point of view on the ongoing mental preparation of karateka competing in kata. Based on these experiences, the key mental skills required of kata competitors have been identified, which include: building self-confidence, coping with stress, managing anxiety, maintaining focus of attention and maintenance of executive function (Figure 1). It should be noted that competing in kata is about performing sets of movements to the best of one's ability (15), with each set consisting of a series of coordinated and harmonious fighting techniques performed in set sequences and timings, representing an imaginary fight against opponents (16).

## Mental preparation of a kata competitor

### Building self-confidence

The objective of mental training in self-confidence is to attain a state of mind wherein the karateka possesses self-esteem and confidence in their ability to accomplish tasks. The athlete strives to achieve complete conviction that they possess the knowledge to perform the required actions (17). A lack of self-confidence can result in the loss of competitions or the avoidance of competition, even when the physical preparation is optimal. In developing self-confidence, the athlete's initial focus is on understanding their worth, strengths, and weaknesses, and their potential for further development. Subsequently, they engage in work on inner speech and imaginative training. In this process, the athlete has to have a clear vision of their desired sporting development. One effective exercise to facilitate this is to write a letter to oneself about one's successful future self and to maintain this vision in their sporting performance (18).

In addition, effective visualisation training engages all the senses: hearing, sight, smell, touch and taste. Furthermore, it encompasses the experience of emotional states. The perspective may be external, whereby the athlete visualises themselves performing the kata, or internal, whereby the environment is seen through the eyes of the athlete. Also the angle of perception of the imagined situation can be modified (19). The size, brightness, colour of the image, the volume and pitch of the sound, and the intensity and temperature of the kinaesthetic sensations are also modified in order to select those that will result in a deep experience and the desired state. During visualisation training, the focus is on mentally repeating the karateka's starting strategies depending on the situation, with a sense of maintaining control and confidence in different situations during competition (20).

Furthermore, it is beneficial to ascertain which values are of the greatest importance to a karateka—combat sport, martial art, self-defence system—and how these can be leveraged during training and in competition to attain success (21). It is similarly important to consider the character predispositions and personality of the karateka. Once the natural functioning style of the athlete has been identified, it is essential to ascertain the extent to which their character strengths are utilised in training and competition scenarios, as well as the scope for further development (22). Additionally, an understanding of their personality profile will reveal predispositions that can enhance their effectiveness at each stage of the macro-cycle, meso-cycle and micro-cycle, as well as in situations that may be contrary to their competitive preferences (23).

An additional factor contributing to the development of self-confidence is the process of inner speech, or directed thinking. Thoughts can give rise to several risks, including mispredicting the future, misexplaining the intentions of others, or misreading situations. As a consequence, thoughts may become irrational, distorted, biased and dysfunctional (24). Consequently, thoughts may serve as a catalyst for, or a foundation of emotional and behavioural difficulties. Thus, training in the reformulation of negative thoughts must begin with the identification of such thoughts. Here, a chart can be employed, in which the athlete records their negative sports-related thoughts that occur during the day. The thoughts are attributed to the situation in which they occurred, together with emotions and behaviour. For each thought, the facts that support and contradict it are identified. Then, on the basis of the facts written out, the athlete attempts to recognise the accuracy of the thought. Subsequently, the thought must be reformulated into one that is consistent with reality, devoid of the above errors (25).

### Coping with stress

It is essential that each karateka experiences a certain degree of stress to ensure effective readiness to compete (26). Only when the threshold for achieving this readiness is exceeded, stress becomes harmful and exhausts the body (27). At the outset of working with an athlete, it is beneficial to ascertain the areas in which they experience the greatest and least stress, which may be suitable for further exploration. Such factors as the coaching staff, rivals, training, taking part in competitions, the presence of the public at competitions, and relationships with fellow karate club members should be taken into account. Subsequently, the karateka identifies which aspects of their situation they perceive to be within their control. Stress management styles of different karateka may vary considerably and should not be imposed. These styles can be broadly categorised as task-oriented, emotion-focused, avoidance-based or will-based, and they are typically associated with specific types of stressful situations. In light of this, the sport psychologist and the athlete in question should undertake a detailed examination of the stressful situations that the athlete encounters, trying to identify and implement effective support strategies (28).

Stress is linked to a variety of physiological responses, including muscle tension, elevated heart rate and respiration, and a sensation of coldness in the extremities. By being able to influence these physiological reactions, one can counteract the negative effects of stress. Two techniques, often performed in parallel, are beneficial in managing stress and its associated physiological responses: Jacobson's progressive relaxation and Schultz's autogenic training (29). Progressive relaxation teaches the athlete to distinguish between muscle tension and relaxation, enhancing their body awareness. Autogenic training elicits the physiological responses associated with a relaxed state. In instances where the karateka is experiencing a high level of stress or a state of stress overload, the mental training regimen should be realigned with biofeedback (30). A strategy for managing stress in a competitive setting is the pre-start routine. In collaboration with the karateka, the sports psychologist develops routines and scripts comprising the scheduling of a physical and mental warm-up period, the avoidance of distractions, the regulation of time prior to the commencement of the kata, and the performance of activities that the athlete finds conducive to optimal performance (31).

### Managing anxiety

Anxiety, as a component of neuroticism, is associated with the formation of a negative cognitive representation of past or future events (32). Given the correlation between anxiety levels and a lack of focus on the present, mindfulness techniques have been demonstrated to be effective. The research results indicate that the implementation of long-term, regular mindfulness training can significantly reduce anxiety levels in athletes (33). Another efficacious technique for the management of anxiety in kata athletes is behavioural de-escalation. This involves the determination of the intensity of the stimulus, in the case of pre-competition anxiety. The work commences at an intensity level below this threshold. A given stimulus is combined with a sensation that is pleasant for the karateka, with gradual increase in intensity (34).

### Maintaining focus of attention

The concept of attention, understood as a set of interacting brain processes active during cognitive actions, plays an integral role in mental preparation in karate (35). The processes that are of particular significance here are the capacity to actively perceive stimuli, the ability to sustain attention from the outset to the conclusion of a kata, vigilance, the selectivity of stimuli, concentration on the task at hand, the ability to switch attention between tasks, and the capacity to control attention between tasks (36). Furthermore, attention can be classified into distinct styles. These include broad and narrow external attention, as well as broad and narrow internal attention. Training in external wide attention enables the karateka to adeptly discern the various stimuli present in their environment, whereas external narrow attention facilitates the concentration on a selected aspect (37). Consequently, internal attention is manifested in the capacity to create analyses and strategies based on experience, and to discern one's own thoughts and feelings before and during the execution of movements, is contingent upon the combination of internal and external attention. This enables the adjustment of the optimal strategy for the execution of the kata (38).

Consequently, in the mental preparation of the karateka, a series of exercises are conducted with the objective of fostering long-term focus, disregarding distractors and optimising focus for a brief period. These exercises can be integrated with physical training, during which the athlete develops the ability to focus on a single signal at a time: the athlete is required to perform kata in the presence of distractors (39). The exercises should be initiated at a level the athlete is capable of mastering. Then, the level of difficulty should be augmented by increasing the number of distractors. An efficacious technique to enhance the maintenance of attention is for the karateka to establish a set of mantras or affirmations that can be employed in instances of lapses or distractions (40).

### Maintenance of executive functions

Executive functions may be defined as the ability to transition from purposeful thinking through action to the realisation of that purpose. These abilities include planning, control, and flexibility of action, initiative, self-regulation and inhibition of reactions, and insight (3). From the perspective of karate competition, all of these competencies are of equal importance in the mental preparation of the kata competitor. They can be exercised through tasks that require switching from one principle or reaction to another, tasks that present a conflict with the possibility of interference from instructions, tasks that require the inhibition of reflexive reactions, or tasks that aim to solve complex problems (41). Additionally, tasks based on the Stroop Effect, which measure reaction time in the presence of name and colour interference, have also been shown to be effective (42).

## Discussion

The modern sport psychology provides many methodologies, techniques, instruments, and resources for working with athletes. This enables the identification of the constituent elements of mental preparation for a given sport discipline, and for a specific sport competition. However, the role of a mental coach and sport psychologist differs somewhat, as it is contingent upon the competencies that are legally permitted (43).

A mental coach works with athletes who function well in society and can apply the skills and strategies they have developed in a competitive sporting environment to other areas of their lives. In this case, the work of the mental coach involves the systematic training of the mental faculties of karateka, aiming at developing their potential, self-confidence, sense of fulfilment and satisfaction (44). The mental coach's work is oriented towards the present and the future, with the objective of enhancing the karateka's inner potential and improving their resources. It is also a partnership-based collaboration (45). A sport psychologist, conversely, possesses the competencies of both a mental coach and a psychologist, and is bound by ethical standards. She is therefore engaged in the provision of mental training, diagnosis, counselling and intervention services to athletes. In addition to mental preparation, the sport psychologist provides support in daily functioning in sport and personal life, applying methodologies from psychology (46). Furthermore, the sport psychologist may also be involved in training for karate coaches and referees and in research in this area. In contrast to a mental coach, a sport psychologist also works with athletes who are experiencing mental health difficulties (47). In such cases, working with karateka addresses their resistance, emotional suffering, dysfunction and working through past traumatic experiences. In recent years, a new specialisation in this field, clinical sport psychology, has been established (48).

In light of the aforementioned issues, the behaviour, personality and intelligence quotient of a kata competitor should be verified through an interview at the outset of their involvement and further verification should be conducted through psychological assessment and interview. However, these measures are the competence of a sports psychologist, who can then effectively determine the individual predispositions of the kata competitor—their needs and potential. In this regard, a sports psychologist is at an advantage over the mental coach, who is constrained to interviews and non-psychological tests, as their availability is not limited to experts in the field (49). The mental preparation of the kata competitor begins at the initial level and progresses to increasingly challenging tasks, facilitating a learning effect (50). At a more advanced stage of mental skill mastery, physical training should be integrated with mental training. This comprehensive approach to psycho-physical preparation reflects competitive situations and considers the distinctive aspects of kata competition (51).

### Practical recommendations

Abstracting from undertaking crisis intervention and providing psychological assistance, which are specific only to sports psychologists, practical recommendations for mental preparation are provided. A mental coach or a sport psychologist preparing a kata competitor for a sporting competition must possess a comprehensive understanding of karate as a sport, including an in-depth knowledge of the specifics of kata competition. Mental preparation is an ongoing process. This indicates that the mental coach or a sports psychologist should collaborate with the athlete throughout the macro-cycle. Mental preparation for a kata competitor should commence several weeks prior to the planned macro-cycle. This allows for a comprehensive assessment of the competitor's abilities and potential in a controlled and non-pressurised environment. The focus of the mental preparation will be distributed throughout the macro-cycle in consideration of the scheduled competitions, the psycho-physical availability of the athlete, and the monitoring of their performance. It is also crucial for the mental coach or sports psychologist to be able to collaborate effectively with the training staff. In a professional team, this comprises a coach, a technical coach, a motor preparation coach, a physiotherapist, a doctor, a physiologist, a nutritionist, a statistical analyst and a manager. Also, professional experience within a sporting environment plays a significant role in the work of a mental coach or sports psychologist.

It is therefore recommended that the experience of a mental coach or sports psychologist from other sports be employed in the mental preparation of a kata competitor. This is a positive value in favour of the mental coach or sport psychologist, as it increases their ability to conduct mental preparation. The final point to be addressed is the obtaining of voluntary consent from the kata athlete for mental preparation. Without this, it is impossible to implement the process described in this article. At the same time, it should be noted that a mental coach is hired only for mental preparation. And a sports psychologist is recommended for broader work with a karateka, beyond mental preparation. Nevertheless, there are also cases of kata athletes forming their psychological skills solely with a karate coach.

### Limitations of the perspective

This perspective is the first article on the mental preparation of kata athletes. Yet, the derived regularities are based on the experience of the Polish karate environment, in which most of the karateka are associated with Olympic karate, Kyokushin and Shotokan styles.

## Conclusions

The presented mental preparation is universal for all kata competitors. However, their individual predispositions may translate into the use of other mental training techniques. Therefore, further research explorations are advisable.

## Data Availability

The original contributions presented in the study are included in the article/Supplementary Material, further inquiries can be directed to the corresponding author.
